# A Fourth Year in the Medical School

**Published:** 1880-05

**Authors:** James C. White

**Affiliations:** Med. Dept. Howard Univ’y.


### Selections.

---

A FOURTH YEAR IN THE MEDICAL SCHOOL. By Professor JAMES C. WHITE, M.D., Med. Dept. Howard Univ'y.

Ten years have not yet passed since the medical department of the University took that great step which has so largely changed the character of medical education in this country, and each succeeding year has demonstrated more positively the advantages of the measures then adopted. The classes have grown constantly larger; and the quality of the students has improved in the same degree, nearly one-half of them being at present graduates in arts or sciences. The complete success of this reform has not failed to impress itself upon other medical schools in all parts of the country, and some of the most important of them have adopted and are about to adopt the "Harvard plan." In the meantime the school has not been content to rest upon the merits of its first step. As soon as practical it instituted an admission examination, so as to exclude at the start that class of students who would approach the study of medicine unfitted by any previous mental training; and it has just now materially added to the efficiency of this preliminary test. It has continuously sought to enlarge the scope, to systematize and to improve the quality, of its instruction, and has been constantly adding to its corps of instructors, which now number over forty. But with this great increase in the amount of teaching, a serious obstacle arose—a want of time in which to teach properly. It had become, in fact, an impossibility for the student to profit by all the instruction offered; and some of the special branches taught, those not considered essential in the requirements of graduation, were largely and necessarily neglected.

The medical faculty has long recognized the necessity of adding another year to the curriculum. America is the only civilized country which gives the degree of doctor in medicine in a three-years' course of study. In how much less time, and on what slight requirements, many of her colleges confer this title, I am ashamed to say. Several of the European universities require six years of study. But the establishment of a fourth year was felt to be also a serious matter in the face of the strong rivalship of schools so lax in their requirements in this regard; and it has not seemed practicable before the present full financial prosperity of the school was assured, and its extensive system of general and special teaching thoroughly organized, to attempt this additional step in medical education. Even now the faculty does not deem it wise to make the four-years' course obligatory at the start, but has left it optional for the present to the student, whether he will continue to attempt to crowd an impossible amount of work into three years, or spread his studies over the longer period in a proper order, and reap the full advantages of all the instruction which is offered him. There can be no doubt that the latter course will be chosen by that large class of students who are constantly disappointed in their endeavors to do justice to all the teaching open to them. To those, however, who are unable to avail themselves of this extended plan of education, the degree of Doctor of Medicine will continue to be given, until further notice, at the end of three years of study under the conditions hitherto observed, and there will be no diminution in the amount, or change in the arrangement, of instruction hitherto given in this course.

The faculty, however, urgently recommend the following plan of study to all students desiring a thorough medical education:

FIRST YEAR.—The studies of the first year are to be, as hitherto, anatomy, physiology and general chemistry; and there will be an examination upon these at the end of the year.

SECOND YEAR.—During the second year the studies will be topographical and practical anatomy, pathological anatomy, medical chemistry, and materia medica; and an examination will be held upon them at the close of the year, general anatomy excepted.

THIRD YEAR.—At the end of the third year there will be an examination in therapeutics, theory and practice, obstetrics, and surgery; and at the close of the

FOURTH YEAR, in clinical medicine, clinical and operative surgery, obstetrics, clinical and operative obstetrics, ophthalmology, otology, dermatology, syphilis, mental and nervous diseases, laryngology, hygiene, legal medicine, diseases of women, and diseases of children; but the main studies of the third and fourth years will be more or less continuous.

The instruction in the special branches, in which an examination is now for the first time instituted, is intended to be more clinical and individual in character than that heretofore given, and should in a large measure take the place of that practical private teaching which has been hitherto sought by American students in European schools after graduation. In the general branches of medical education, too, instruction will be carried, especially in practical directions, farther than was possible in the former cramped period of study. The degree of Doctor of Medicine will be given to candidates who have passed a satisfactory examination in all the studies of the four-years' course, and the distinctive degree *cum laude* to those who have pursued the whole course, and have obtained an average of seventy-five per cent. upon all the examinations above given.

The new plan of instruction goes into operation at the beginning of the next academic year, and can be adopted by all students who are then members of the school. It deserves, and will undoubtedly receive, the earnest support of the profession in all parts of the country.

---

HEMORRHOIDS TREATED WITH CAPSICUM.—Vidal. (*Journ. de Méd.*, Feb., 1880, p. 70.)

In cases of hemorrhoidal congestion Vidal regards capsicum annuum as the best remedy. He prescribes four or five pills daily, each containing 20 centigrams, half at breakfast-time and half at supper-time. Under this influence the congestion and all the painful symptoms which accompany it disappear rapidly.

